# Calcaneal Tuberosity Fracture With Complete Achilles Tendon Rupture: A Unique Surgical Challenge

**DOI:** 10.7759/cureus.57914

**Published:** 2024-04-09

**Authors:** Phillip A McCarthy, Sohaib Shah, Dev Thakker, Lee David

**Affiliations:** 1 Trauma and Orthopaedics, Maidstone and Tunbridge Wells National Health Service (NHS) Trust, Royal Tunbridge Wells, GBR

**Keywords:** calcaneus reconstruction, tendo achilles rupture, achilles rupture, achilles tendon injury, calcaneus fractures

## Abstract

A calcaneal tuberosity avulsion fracture occurring simultaneously with a rupture of the Achilles tendon, although occurring through similar mechanisms, is a rare injury pattern to see in combination and presents a unique challenge to the surgeon. The patient we present was initially found to have a type II fracture of the calcaneal tuberosity. However, during surgical fixation of the fracture, a complete rupture of the Achilles tendon was noticed.

The technique used in this case was the fixation of the fracture fragment with two 5 mm fully threaded screws. The tendon was then reattached to the calcaneus using two Mitek anchors (DePuy Mitek Inc., MA, USA) with a modified Bunnell technique. There are a number of techniques suggested in the literature, including, among others, K-wires (DePuy Mitek Inc., MA, USA) and screw fixation. Our patient recovered well and has now been discharged from further orthopaedic follow-up.

## Introduction

Avulsion fractures of the calcaneal tuberosity are rare, constituting about 3% of all calcaneal fractures. They usually occur as a result of a fall with the ankle in plantarflexion, ankle hyperextension, or a sudden muscle contraction while the foot is planted on the ground. These fractures have been further classified into types I-IV by both their mechanism and which fibres of the Achilles insertion are affected. The type II fracture that our patient presented with has a high risk of skin tenting and necrosis associated with it and therefore may need to be reduced and fixed as an emergency [1].

Achilles tendon rupture, however, is the most common tendon rupture in the lower extremity, occurring in 6-18% of athletes [2]. The main causal associations for rupture are direct trauma or forced dorsiflexion in a patient who is not regularly active with a medical background causing tendinopathy. A strong association was found with diabetes as well as osteoporosis, renal disease, and hyperparathyroidism [3]. Achilles tendon rupture can be managed either non-operatively with an early, closely monitored return to function or operatively [4]. Meta-analysis has shown a lower rate of re-rupture with operative management but a higher risk of complications such as infection [5,6].

A calcaneal tuberosity avulsion fracture occurring simultaneously with a rupture of the Achilles tendon rupture, although occurring through similar mechanisms, is a rare injury combination indeed and produces a unique surgical challenge. Without proper surgical reduction and fixation of the fracture fragment with repair of the tendon, significant dysfunction could result. Intra-operative discovery of this complication also requires quick adaptation to prevent a prolonged operation resulting in increased tourniquet time and patient morbidity. This case report will describe a patient with a calcaneal avulsion fracture with simultaneous Achilles tendon rupture and the novel surgical approach to repair both.

## Case presentation

A 59-year-old male patient presented with a painful and swollen right ankle after having tripped down a ditch near his carp pond. He was unable to recall the precise movement of his ankle during the fall but reported that his hindfoot was immediately painful and became swollen shortly afterward. He had been unable to weight-bear since the injury. He had never smoked, took no regular medications, and had no significant past medical history. He had presented to the hospital immediately following his injury.

**Figure 1:**
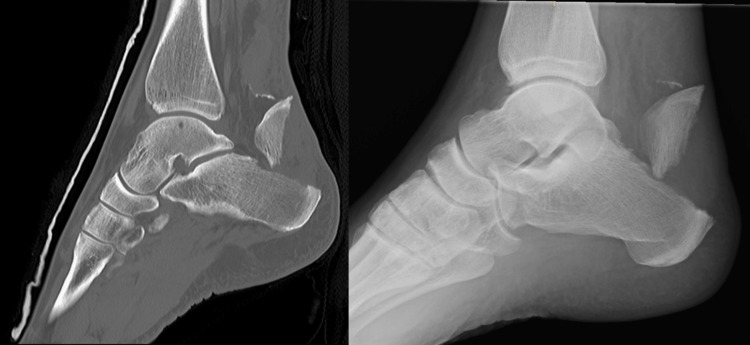
A sagittal CT (right) and lateral X-ray showing the fracture fragment and further small avulsion fragment CT: computed tomography

An initial plain film radiograph showed a type II fracture of the calcaneal tuberosity. Prior to placing the patient in an equinus cast, the skin overlying the fracture fragment was examined to ensure that it was not compromised. Due to the initial examination demonstrating a palpable gap proximal to the calcaneus, an ultrasound scan was also performed to assess the integrity of the Achilles tendon. The report showed no evidence of a rupture of the Achilles tendon. There was evidence of avulsion of a bone spur just proximal to the fracture fragment seen on both the plain film and CT (Figure 1).

The patient was taken to the theatre for fixation of the fracture of the calcaneus. Due to the integrity of the posterior skin, this was done on the next urgent orthopaedic list the next day. The intention was to reduce and fix the fracture percutaneously; therefore, the fracture was reduced with the aid of fluoroscopic guidance through a small posterolateral stab incision.

**Figure 2:**
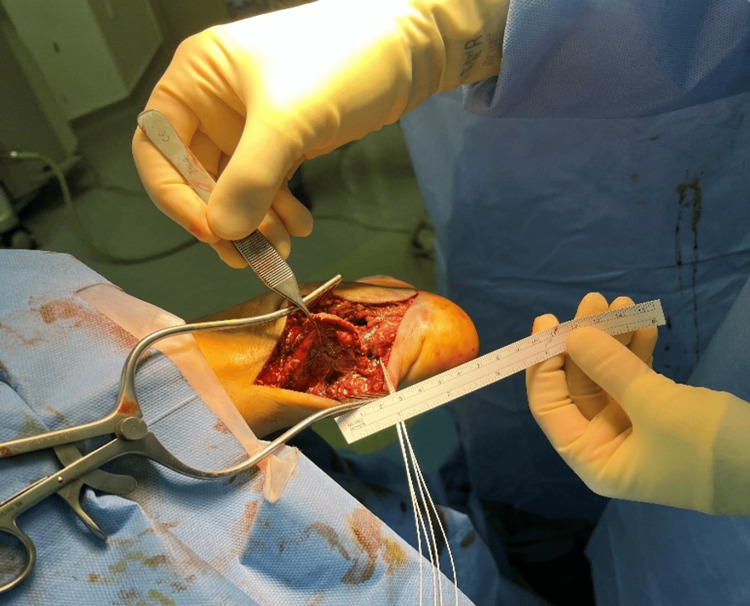
Images taken intra-operatively show the avulsed end of the Achilles tendon held by forceps. The sutures from the Mitek anchors can be seen. Imaging was used with the patient’s signed permission

On reduction of the fracture, the Achilles tendon could not be palpated, with a gap palpable over the posterior ankle. The incision was extended to visualise the tendon, and it was seen to have completely avulsed from the fracture fragment, taking with it small fragments of bone (Figure 2). The decision was made to first fix the calcaneal tuberosity fragment using two 5 mm compression FT screws (Arthrex Inc., Munich, Germany). The attention was moved to reattaching the ruptured Achilles tendon. As there was no tendon remnant to which to tie the Achilles, two Mitek anchors (DePuy Mitek Inc., MA, USA) were fixed to the calcaneal tuberosity and sutured to the tendon using a modified Bunnell technique. The MiTek anchors were fixed perpendicular to the direction of force to maximise their pull-out strength. The sutures were tied to the ankle in plantar flexion, and the fixation was tested. The patient was then placed in a backslab in plantarflexion and advised to follow a non-weight-bearing rehabilitation protocol regarding his lower extremity injury.

**Figure 3:**
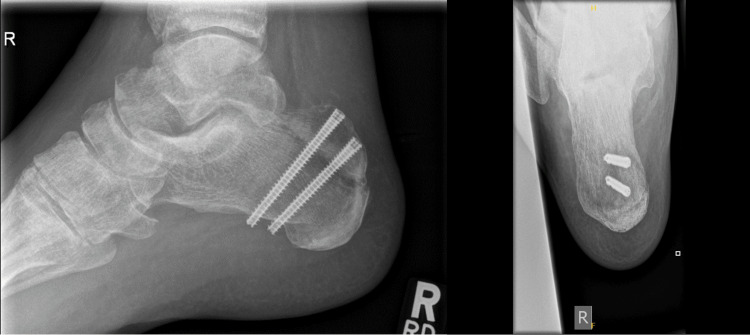
Final plain film radiographs taken twelve weeks post-operatively show an almost complete fracture union

On discharge from the hospital, our patient was advised not to weight bear on the affected side in a VACOcast walking boot (OPED Medical Inc., Melksham, UK). At two weeks post-op, this was changed to an Aircast (DJO Global, Texas, USA) with three wedges in the heel. At six weeks post-op, he began physiotherapy to increase the range of movement of the ankle and was able to partially weight bear on the foot. He was finally reviewed twelve weeks following his operation, and apart from some limitations in dorsiflexion of the ankle, there were no concerns regarding his recovery, and the final plain film radiographs showed an almost complete union of the fracture (Figure 3). The decision was made for him to be discharged from the regular orthopaedic follow-up to continue his rehabilitation under the physiotherapy team.

## Discussion

A case where a calcaneal tuberosity fracture occurred alongside an Achilles tendon rupture, to our knowledge, only appears once in the literature [7]. In that case, the approach taken was, once the calcaneal tuberosity fragment was fixed using two 7.3 mm cannulated screws, FiberWire (Arthrex Inc., Munich, Germany). was stitched into the Achilles tendon and then threaded through the screws to the plantar aspect of the calcaneus. Here, the two threads were tied over a padded button. The sutures were cut, and the button was removed at six weeks post-fixation. At six months, the patient was fully weight-bearing and back to normal functioning. This was a modification of a technique proposed by Squires et al. [8] for the fixation of a small calcaneal avulsion fracture where sutures tied to the Achilles tendon were passed through bone tunnels drilled into the calcaneus and tied over the plantar aspect. With this technique, there is a concern that, with the button placed on the plantar aspect of the foot, there would be a risk to the soft tissues, which would increase the risk of plantar fasciitis. Additionally, as with the above technique, the patient required a further procedure to cut the suture ends and remove the button tie.

With regard to the fixation of calcaneal tuberosity avulsion fractures, there are a number of surgical options available for fixation. Most commonly, a lag or compression screw is used to fix a large bony fragment. However, techniques using K-wires (DePuy Mitek Inc., MA, USA) and tension band wiring have also been suggested [9], although the bulky construct can cause local soft tissue complications. The technique described by Squires et al. [8] can also be used in conjunction with lag screws to capture larger fragments.

MiTek anchors are currently employed in a variety of operations, from rotator cuff repair to repositioning of the temporomandibular joint disc. Their high pull-out strength has also been utilized in the repair of quadriceps and Achilles tendon avulsions [10]. In this case, although the anchors were fixed into the fracture fragment and so increased the risk of displacement, the risks of soft tissue injury from placing suture ties on the plantar aspect of the foot were aimed at being reduced.

## Conclusions

Despite this being a rare presentation, this injury shows the challenge to the surgeon to best utilise the tools and skills available to them to ensure a good outcome for the patient. From reviewing the literature, there is no one gold standard method indicated in this injury pattern but rather a number of proposed methods using a range of equipment available to the orthopaedic surgeon.

In this case, we have shown a novel approach that has provided adequate fixation and therefore ensured a satisfactory outcome for the patient in an unexpected intra-operative finding. We hope that this report will demonstrate the efficacy of this method of fixation for those confronted with this unusual challenge.

In addition to the surgical fixation of this injury, care must also be taken to keep your patient informed, as his recovery will need to be closely monitored to ensure that either fixation of the Achilles tendon or calcaneal tongue does not fail. In this case, the patient was only followed up routinely for 12 weeks post-operatively and only had plain film radiographs to confirm the continued hold of the fixation. Although he reported no concerns prior to discharge from follow-up, due to the significant soft tissue injury sustained during his injury and subsequent surgical repair, it may have been prudent to follow up for a longer period to confirm complete bony union.
